# Antibiotic treatment-induced dysbiosis differently affects BDNF and TrkB expression in the brain and in the gut of juvenile mice

**DOI:** 10.1371/journal.pone.0212856

**Published:** 2019-02-22

**Authors:** Michela Bistoletti, Valentina Caputi, Nicolò Baranzini, Nicoletta Marchesi, Viviana Filpa, Ilaria Marsilio, Silvia Cerantola, Genciana Terova, Andreina Baj, Annalisa Grimaldi, Alessia Pascale, Gianmario Frigo, Francesca Crema, Maria Cecilia Giron, Cristina Giaroni

**Affiliations:** 1 Department of Medicine and Surgery, University of Insubria, Varese, Italy; 2 Department of Pharmaceutical and Pharmacological Sciences, University of Padova, Padova, Italy; 3 APC Microbiome Ireland, University College Cork, Cork, Ireland; 4 Department of Biotechnology and Life Sciences, University of Insubria, Varese, Italy; 5 Department of Drug Science, University of Pavia, Pavia, Italy; 6 Department of Internal Medicine and Therapeutics, Section of Pharmacology, University of Pavia, Pavia, Italy; Chiba Daigaku, JAPAN

## Abstract

Antibiotic use during adolescence may result in dysbiosis-induced neuronal vulnerability both in the enteric nervous system (ENS) and central nervous system (CNS) contributing to the onset of chronic gastrointestinal disorders, such as irritable bowel syndrome (IBS), showing significant psychiatric comorbidity. Intestinal microbiota alterations during adolescence influence the expression of molecular factors involved in neuronal development in both the ENS and CNS. In this study, we have evaluated the expression of brain-derived neurotrophic factor (BDNF) and its high-affinity receptor tropomyosin-related kinase B (TrkB) in juvenile mice ENS and CNS, after a 2-week antibiotic (ABX) treatment. In both mucosa and mucosa-deprived whole-wall small intestine segments of ABX-treated animals, BDNF and TrKB mRNA and protein levels significantly increased. In longitudinal muscle-myenteric plexus preparations of ABX-treated mice the percentage of myenteric neurons staining for BDNF and TrkB was significantly higher than in controls. After ABX treatment, a consistent population of BDNF- and TrkB-immunoreactive neurons costained with SP and CGRP, suggesting up-regulation of BDNF signaling in both motor and sensory myenteric neurons. BDNF and TrkB protein levels were downregulated in the hippocampus and remained unchanged in the prefrontal cortex of ABX-treated animals. Immunostaining for BDNF and TrkB decreased in the hippocampus CA3 and dentate gyrus subregions, respectively, and remained unchanged in the prefrontal cortex. These data suggest that dysbiosis differentially influences the expression of BDNF-TrkB in the juvenile mice ENS and CNS. Such changes may potentially contribute later to the development of functional gut disorders, such as IBS, showing psychiatric comorbidity.

## Introduction

Numerous studies have established that the naturally occurring commensal microbiota, which in the human gut is composed of about 3.8X 10^13^ bacterial cells belonging to approximately 2000 species, represents an essential organ for the host homeostasis by contributing to the metabolism of nutrients, development of the immune host defence and maturation of the gastrointestinal (GI) tract [1]. The complex array of cellular elements constituting the enteric microenvironment (enterocytes, enteroendocrine cells, immunocytes, smooth muscle cells, interstitial Cajal cells, enteric neurons and glial cells) responds to microbial factors, mainly via pattern recognition receptors (e.g. Toll-like receptors, TLRs) [2,3], neurotransmitter, neuropeptides and neurohormone receptors [4–6]. The commensal gut microbial flora influences the development and function of the enteric nervous system (ENS), which consists of two complex interconnected neuroglial networks, constituting the submucosal and myenteric plexus [7]. Microbiota-induced neuronal plasticity is, however, not limited to the ENS but can potentially activate responses in the central nervous system (CNS), via activation of neuroendocrine and metabolic pathways, along the microbiota-gut-brain axis [8]. Several preclinical studies, carried out in animals fed with specific dietary regimens and in transgenic animals or germ-free (GF) rodents, have shown that dysfunction of the gut microbiota during early life (infancy, childhood and adolescence) has important consequences, not only on the normal gut functions, but also on the brain and behaviour, including pain perception, stress response and anxiety [9]. During adolescence neurons undergo crucial structural, neurochemical and molecular changes in response to genetic and environmental signals both in the CNS and in the ENS [10,11]. During this stage of life, the symbiotic microbial flora experiences dynamic processes, such as changes in the composition and relative abundance of various microbial constituents, which may influence neuronal development [11,12]. Environmental insults, such as the use of antibiotics, stress, harmful events and poor diet, may result in dysbiosis-induced neuronal vulnerability both in the ENS and CNS. This neuronal susceptibility to microbiota alterations contributes to the onset of chronic functional GI disorders, such as irritable bowel syndrome (IBS), which is associated with psychological distress and psychiatric comorbidity, including depression [9, 13]. From this perspective, it is important to evaluate whether changes in the symbiotic microbial flora during adolescence may influence the expression of some factors involved in neuronal development and plasticity both in the ENS and CNS. In this study, we have focused on the role of brain-derived neurotrophic factor (BDNF) and its high affinity receptor tropomyosin-related kinase B (TrkB). BDNF plays a central role in promoting neuronal survival and growth, synaptic plasticity and reinforcement of synaptic communication [14,15]. In the CNS, the neurotrophin is a key molecule influencing mood, behaviour and cognitive functions, such as learning and memory, and any alteration of its levels are related to development of psychiatric disorders, such as anxiety and depression [16,17]. The normal gut microbiota influences the expression of BDNF in brain regions crucially involved in the development of correct behavioral patterns, such as the hippocampus and cortex [18,19]. A relationship between development of behavioral disorders, such as anxiety and cognitive deficits, and altered BDNF levels in different CNS regions was demonstrated both in GF mice and in adolescent mice after antibiotic treatment-induced dysbiosis [20–22]. In the GI tract, BDNF is released from different cell types, including enterocytes, enteric glial cells and neurons and plays a fundamental role in modulating both sensory and motor functions [23–26]. BDNF represents a neurotransmitter/neuromodulator in enteric neuronal circuitries, favouring the local release of enteroendocrine molecules and neurotransmitters from sensory and motor neurons, enhancing peristalsis and gut motility both in laboratory animals and in humans [23,24,27,28]. The neurotrophin plays also a modulatory role on visceral pain perception, as demonstrated in studies carried out on rat models of IBS showing that BDNF may favour the development of visceral hypersensitivity by activating both intrinsic and extrinsic primary afferent neurons [25,29]. In addition, BDNF released from dorsal root ganglia and spinal cord contributed to the development of exaggerated visceromotor responses to colorectal distension in a rat model of colitis [30]. In spite, of the central role played by BDNF in the microbiota-gut-brain axis, there are no reports, at least up to our knowledge, concerning the occurrence of BDNF and TrkB expression changes in the ENS, caused by alterations in the gut commensal microflora during adolescence. The demonstration of such relationship would be, even more, interesting since, in the ENS of juvenile mice, important rearrangements of both excitatory and inhibitory neurotransmitter pathways regulating intestinal motility occur after antibiotic-induced dysbiosis [31]. Thus, the aim of this study was to evaluate the expression of BDNF and TrkB in the ENS of juvenile mice after a massive chronic antibiotic treatment, by means of morphological and molecular biology approaches. In view of the ability of the gut microbiota to influence neurotrophin expression in the CNS, we also investigated BDNF and TrkB levels in the hippocampus and prefrontal cortex.

## Materials and methods

### Animals

Juvenile male C57BL/6J mice (3±1 week-old, body weight 18±1 g, Charles River Laboratories, Italy) were housed in groups of four animals in individually ventilated cages with sawdust on bottom, under controlled environmental conditions (temperature 21±1°C; relative humidity 60–70%). Animals had free access to a standard laboratory chow and tap water and were maintained at a regular 12/12-h light/dark cycle. Animal care and handling were in accordance with the European Union Council Directive 2010/63, recognized and adopted by the Italian Government (Decree No. 26/2014). The protocol was approved by the Animal Care and Use Ethics Committee of the University of Padova and University of Insubria and by the Italian Ministry of Health (Ref n. 1142/2015-PR).

### Animal treatment

Gut dysbiosis was induced in adolescent mice according to a previously described pharmacological procedure, which reproduces a germ free-like phenotype [31]. Briefly, a cocktail of broad-spectrum antibiotics (50 mg/kg vancomycin, 100 mg/kg neomycin, 100 mg/kg metronidazole and 100 mg/kg ampicillin, ABX-treated mice group), was administered to conscious mice every 12 hours for 14 days by oral gavage (100 μl/mouse), using a stainless-steel feeding tube [31]. Tap water was administered as vehicle to control (CTR) mice. Animals were randomized to treatment groups. To normalize gut microbiota, mice colonies from both groups were housed in the same room and generally in the same cages and maintained by the same personnel. At the end of procedures, animals were euthanized by cervical dislocation. The small intestine was removed and washed with a physiological Krebs solution (composition in mM: NaCl 118, NaHCO_3_ 25, glucose 11, KCl 4.7, CaCl_2_·2H_2_O 2.5, MgSO_4_·7H_2_O 1.2, K_2_HPO_4_ 1.2). For western blot and qRT-PCR analysis, experiments were carried out separately on the mucosa and mucosa-deprived whole wall segments. Western blot and qRT-PCR analysis experiments were carried out on the prefrontal cortex and hippocampus. Tissues collected for western blotting and qRT-PCR analyses were kept at -80°C until the experiments were performed. For immunofluorescence staining intestinal segments, prefrontal cortex and hippocampus were fixed immediately after collection.

### Immunofluorescence

The immunolocalization of BDNF and TrkB was performed on paraffin-embedded prefrontal cortex and hippocampus sections and on longitudinal muscle myenteric plexus (LMMP) whole-mount preparations obtained from the small intestine of ABX-treated and CTR mice.

#### Paraffin sections

The prefrontal cortex and hippocampus were dissected and postfixed at 4°C in 4% paraformaldehyde in phosphate buffer: (PBS composition in mM: 0.14 NaCl, 0.003 KCl, 0.015 Na_2_HPO_4_, 0.0015 KH_2_PO_4_, pH 7.4) for 4 hours. Brain regions were treated for 72 h in sucrose 30% v/v in PBS and embedded in paraffin. Cross sections (7 μm) of both ABX-treated and CTR samples were processed as described by Filpa et al. [32]. The details of the primary and secondary antibodies and related optimal dilution are reported in Table 1. PBS buffer used for washing steps and primary antibodies dilutions contained 2% bovine serum albumin (BSA). Control samples were incubated with 2% BSA in PBS. Coverslips were mounted with Citifluor mounting medium and then observed under a fluorescence microscope (Nikon Instruments).

**Table 1:** Primary and secondary antisera and their respective dilutions used for immunohistochemistry (HC) and western blot (WB) assay.

Antiserum	Dilution(WB)	Dilution(HC)	Source	Hostspecies
Primary antisera				
HUC/D, biotin	______	1:100	Invitrogen (A-21272)	Mouse
TrkB	1:100	1: 50	Santa Cruz (sc8316)	Rabbit
TrkB	______	1: 50	Santa Cruz (sc377218)	Mouse
Phospho-TrKB (Tyr816)	1:200	______	Millipore (ABN1381)	Rabbit
BDNF	1:200	1:50	Santa Cruz (sc546)	Rabbit
BDNF	______	1:50	Santa Cruz (sc65514)	Mouse
CGRP	______	1:200	Immunostar (24112)	Rabbit
Substance P	______	1:200	Immunostar (20064)	Rabbit
β-actin	1:1000	_______	Cell Signalling Technology (#3700)	Mouse
Secondary antisera & streptavidin complexes				
Anti-rabbit Alexa Fluor 488	______	1:300	Molecular Probes (A21206)	Donkey
Anti-mouse Alexa Fluor 488	______	1:300	Molecular Probes (A21202)	Donkey
Cy3-conjugated streptavidin	______	1:500	Amersham (PA43001)	______
F(ab’)_2_ Anti-rabbit IgG (H+L) biotin	______	1:300	Caltag laboratories (L43015)	Goat
Anti-rabbit IgG HRP peroxidase conjugated	1:10000	______	Amersham (NA934)	Donkey
Anti-mouse IgG, HRP- linked	1:2000	______	Cell Signalling Technology (#7076)	Horse

**Supplying companies:** Amersham, GE Healthcare, Buckinghamshire, UK; Caltag Laboratories, Invitrogen, Burlingame, CA, USA; Cell Signaling Technology, Danvers, Massachusetts, USA; Immunostar Inc., Hudson, WI; Millipore, Temecula, CA; Molecular Probes, Invitrogen, Carlsbad, CA, USA; Santa Cruz Biotechnology Inc., CA,USA.

#### Whole-mounts

For whole-mount immunolabelling, small intestine segments were fixed with 0.2 mol/l PBS containing 4% formaldehyde and 0.2% picric acid and the longitudinal muscle with the attached myenteric plexus (LMMP) was gently removed from the rest of the intestinal wall as previously described [33]. After blocking nonspecific sites for 2 hours using PBS with added 1% Triton X-100 and 10% normal horse serum (NHS) (Euroclone, Pero, Italy), LMMPs were incubated with optimally diluted primary antibodies (Table 1). Double-labeling was performed during consecutive incubation times: the first primary antibody was added overnight at 4°C, then incubation with the appropriate secondary antibody followed for 2h at room temperature (RT). Preparations were successively incubated overnight at 4°C with the second primary antibody and for 2 h at RT with an appropriate secondary antibody. Preparations were mounted onto glass slides, using a mounting medium (Vectashield; Vector Lab, Burlingame, CA, USA). Total neuron number per ganglion area was expressed as the ratio between the number of HuC/D-immunoreactive (IR) neurons within the ganglion and the total ganglion area (μm^2^) measured with Image J NIH image software (http://imagej.nih.gov/ij). Neuronal cell body area was also measured and analyzed with Image J. Confocal images of 15 ganglia captured from preparations obtained from ABX-treated and CTR groups, respectively, were used both for neuronal cell counting and ganglia area measurement. To establish the proportion of BDNF- and TrkB-expressing myenteric neurons, the number of BDNF-IR or TrkB-IR neurons that co-localized with HuC/D were counted and expressed as percentage of the total number of HuC/D-IR neurons [34]. Since BDNF and TrkB are known to co-localize with some neuropeptides, some experiments were carried out to evaluate a possible co-immunostaining with substance P (SP) or calcitonin related-gene peptide (CGRP) [24,35]. A total of 10–20 ganglia were sampled from LMMP preparations obtained from 5 animals for each experimental group. Negative controls and interference control staining were evaluated by omitting both the primary and/or the secondary antibody, and by incubating colonic whole-mount preparations with nonimmune serum from the same species in which the primary antibodies were raised. No specific signal was detected under any of these conditions. Preparations were analyzed using a Leica TCS SP5 confocal laser scanning system (Leica Microsystems GmbH, Wetzlar, Germany) and pictures were processed with Adobe-Photoshop CS6S software.

### Real-time quantitative RT-PCR

Total RNA was extracted from small intestine mucosa and mucosa-deprived whole wall, prefrontal cortex and hippocampus samples with TRIzol and treated with DNase I (DNase Free) to remove any traces of contaminating DNA. cDNA was obtained by retrotranscribing 2 μg of total RNA using the High Capacity cDNA synthesis kit (Applied Biosystems, Milan, Italy). Quantitative RT-PCR was performed on the Abi Prism 7000 real-time thermocycler (Applied Biosystems, Milan, Italy) with Power Sybr Green Universal PCR Master Mix (Applied Biosystems, Milan, Italy) according to the manufacturer's instructions. Primers were designed using Primer Express software (Applied Biosystems, Milan, Italy) as reported in Table 2 and a final concentration of 500 nM for each primer was used. Primers were designed to have a similar amplicon size and similar amplification efficiency as required for applying the 2^-ΔΔCt^ method to compare gene expression in the ABX-treated group with respect to CTR values [36]. β-actin was used as housekeeping gene. Experiments were performed at least five times for each different preparation.

**Table 2:** Sequence of primers used in the study for the qRT-PCR analysis.

gene		Sequence	
β-actin		F 5’-ACCAGAGGCATACAGGGACA-3’ R 5’-CTAAGGCCAACCGTGAAAAG-3’	
HuC/D		F 5’- AAGAGTCCCCTGTCGCTCA -3’ R 5’- TACACGAAGATGCACCAGCC -3’	
BDNF		F 5’- AACCATAAGGACGCGGACTT -3’ R 5’- TGCAGTCTTTTTATCTGCCG -3’	
TrKB		F 5’- CAGCACCAAGCAGCAAGAG -3’ R 5’- CAAGACCAGCAGGCATAAGC -3’	

### Western immunoblot analysis

Hippocampus, prefrontal cortex, small intestine mucosa and mucosa-deprived whole wall samples were used to analyze BDNF and TrkB protein levels. Briefly, purified membrane fractions were obtained after successive centrifugations, boiled for 5 min in Laemmli sample buffer and processed for electrophoretic separation and blotting as described by Filpa et al. [37]. Membrane incubation with BDNF or TrkB primary antibodies was followed by incubation with a horseradish peroxidase-conjugated secondary antiserum (Table 1). The antibody/substrate complex was visualized by chemiluminescence using an enhanced chemiluminescence kit (ECL advance Amersham Pharmacia Biotech, Cologno Monzese, Italy). Signal intensity was evaluated by densitometric analysis using Image J NIH image software. β-actin and α-tubulin were used as protein loading control for intestinal and CNS areas, respectively. BDNF or TrkB protein levels were expressed as the ratio of the optical density (expressed in arbitrary units) of BDNF and TrkB bands and the optical density of the relevant β-actin or α-tubulin band x1000 obtained in CTR and ABX-treated samples. Experiments were performed at least five times. Negative controls were performed by omitting the primary antibody.

### Statistical analysis

All data are expressed as mean±S.E.M. Statistical significance was calculated with applying the unpaired Student’s t test, using GraphPad Prism (version 5.3 GraphPad software, San Diego, CA, USA). The differences between groups were considered significant when *P* values were < 0.05.

## Results

### BDNF mRNA and protein levels in the mouse small intestine of control and ABX-treated mice

**Fig 1:**
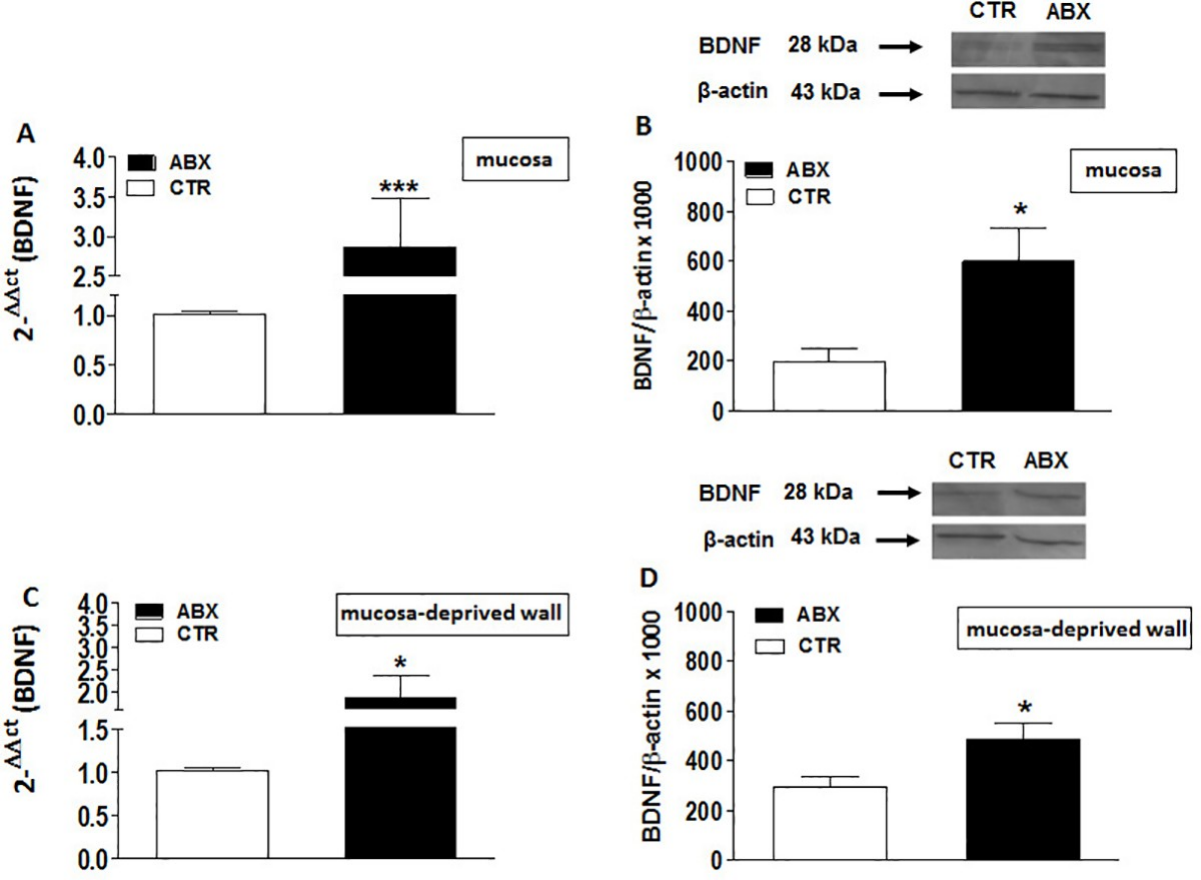
Expression of BDNF in juvenile mouse small intestine after antibiotic treatment-induced dysbiosis. (**A, C**) RT-PCR quantification of BDNF transcripts in preparations of the mouse mucosa (**A**) and mucosa-deprived whole wall (**C**) of the mouse small intestine obtained from control (CTR, empty bars) and ABX-treated (ABX, black bars). Values are mean±S.E.M. of 9 experiments of the percentage variation of relative gene expression with respect to values obtained in CTR animals. The relative gene expression was determined by comparing 2^-ΔΔCt^ values normalized to β-actin. (**B, D**) BDNF protein expression analyzed in the mucosa (**B**) and mucosa-deprived whole wall (**D**) of the mouse small intestine obtained from CTR (empty bars) and ABX-treated animals (black bars). Blots representative of immunoreactive bands for BDNF and β-actin in the different experimental conditions are reported on top of each panel. Samples (200 μg) were electrophoresed in SDS-8% polyacrylamide gels. Numbers at the margins of the blots indicate relative molecular weights of the respective protein in kDa. Values are expressed as mean±S.E.M. of 5 experiments of the ratio x1000 of the optical density (O.D.) of BDNF vs β-actin in CTR and in ABX-treated preparations. *P<0.05 and ***P<0.001 vs CTR animals by unpaired Student’s t test.

qRT-PCR analysis revealed a significant increase of BDNF transcript both in the mucosa (p<0.001) and in mucosa-deprived whole intestinal wall (p<0.05) of ABX-treated mice as reported in panels A and C, respectively, of Fig 1. In mouse small intestine preparations, western blot analysis of BDNF revealed a specific band at 28 kDa. In some samples a double band was also observed, which may represent a post-translational change in the protein [38]. After antibiotic-induced dysbiosis, BDNF protein levels increased significantly both in the mucosal layer (p<0.05) and in the mucosa-deprived whole intestinal wall (p<0.05) (Fig 1, panels **B** and **D**).

### Distribution of BDNF in small intestine LMMP whole-mount preparations from control and ABX-treated animals

**Fig 2:**
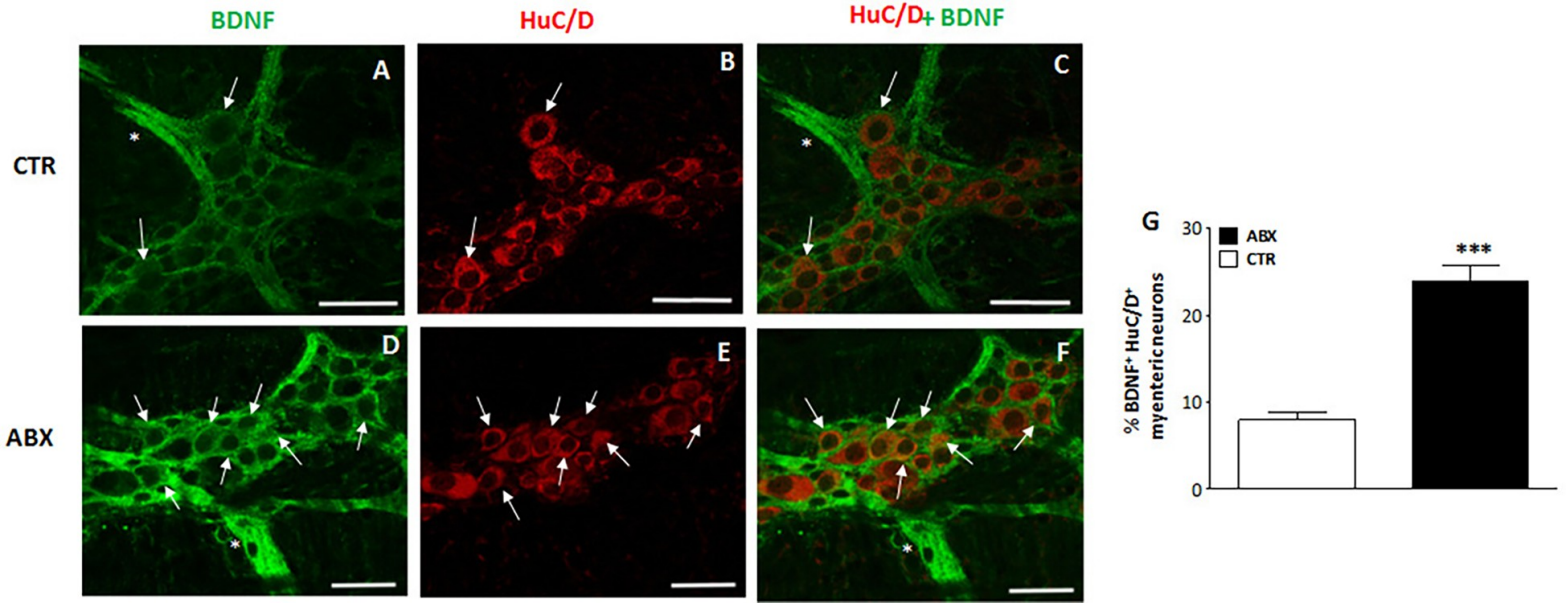
Effect of antibiotic treatment on BDNF distribution in juvenile mouse small intestine LMMP whole-mount preparations. Representative confocal microphotographs showing co-staining of BDNF (green) and HuC/D (pan-neuronal marker, red) in LMMPs obtained from CTR (**A-C**) and ABX-treated mice (**D-F**). In LMMPs obtained from both CTR and ABX-treated mice BDNF stained the soma of both large and medium size neurons with either a round or an ovoidal shape (arrow). BDNF staining was also observed in prolongation surrounding neurons and in the interconnecting fibers between ganglia (asterisk). In CTR preparations, BDNF faintly stained the soma of few neurons. After antibiotic treatment, the number of BDNF positive myenteric neurons was significantly higher than in CTR (**D-E**). Bars: 50 μm. (**G**) Percentage of BNDF immunoreactive myenteric neurons in small intestine of CTR and ABX-treated mice. Values are given as mean ± SEM of at least 20 fields for each intestinal region.***P<0.001 vs CTR animals by unpaired Student’s t test vs CTR.

**Fig 3:**
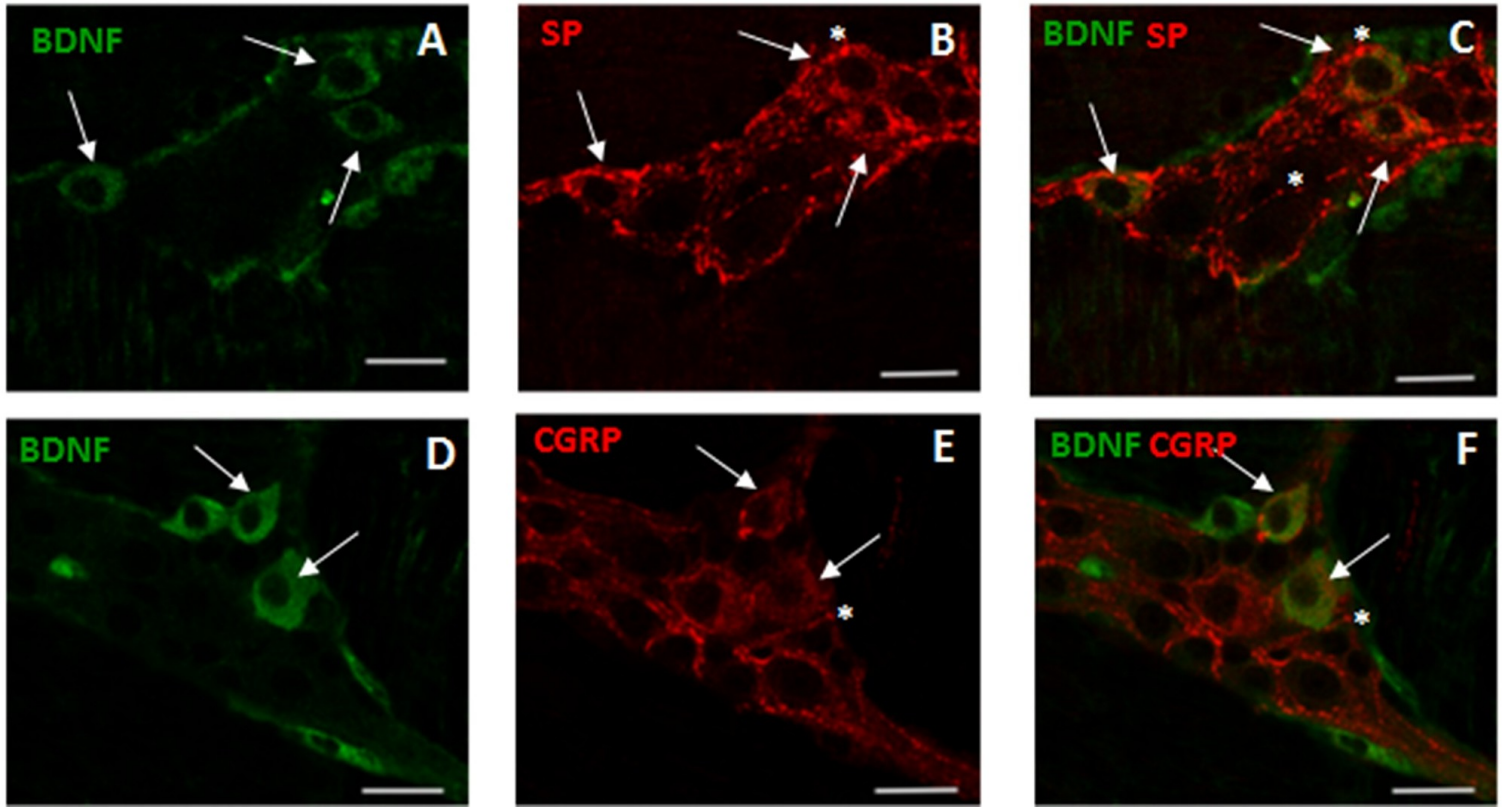
Relationship between BDNF, SP and CGRP immunoreactivity in juvenile mouse small intestine myenteric ganglia after antibiotic treatment. (**A-C**) Representative micrographs of juvenile mouse small intestine LMMP whole-mount preparations obtained from ABX-treated mice showing co-localization of BDNF and SP. BDNF immunoreactivity was found in the soma of a population of SP-IR expressing neurons (arrows). BDNF-IR myenteric neurons were surrounded by varicose SP-IR fibers (asterisk). (**D-F**) Representative micrographs of mouse small intestine LMMP whole-mount preparations obtained from ABX-treated mice showing co-localization of BDNF and CGRP. CGRP-IR was found in the soma (arrows) of BDNF-IR myenteric neurons. BDNF-IR myenteric neurons were surrounded by varicose CGRP-IR fibers (asterisk). Bars: 25 μm.

In control small intestine LMMP whole-mount preparations, BDNF antibody faintly stained the cytoplasm of few myenteric neurons (7.46±0.72%, n = 18) (Fig 2, panel **G**). As observed after costaining with the pan-neuronal marker HuC/D, BDNF antibody stained the soma of large and medium-sized neurons with either a round or an ovoidal shape. In myenteric ganglia, BDNF immunoreactivity was also found in prolongations discontinuously surrounding neurons, in interconnecting strands between ganglia and in bundles of fibers running along the longitudinal smooth muscle (**Fig 2** panels **A-C** and **D-F**). A significant (p<0.0001) increase in the number of BDNF-IR myenteric neurons was observed in ABX preparations with respect to preparations obtained from CTR mice (**Fig 2**, panels **D, F** and **G**). In whole-mount LMMP preparations obtained from both CTR and ABX-treated mice, BDNF-IR neurons co-stained with SP (**Fig 3**, panels **A-C**). Intense SP immunoreactivity was also detected in varicose fibres surrounding myenteric neurons, (**Fig 3**, panels **B-C**). After antibiotic treatment the number of myenteric neurons co-expressing BDNF and SP increased significantly with respect to CTR preparations [51.91±7.52% (n = 13); 10.69±4.97% (n = 12), respectively; p<0.001]. In both CTR and ABX-treated whole-mount preparations BDNF-IR myenteric neurons were also immunoreactive to CGRP (**Fig 3**, panels **D-F**). CGRP immunoreactivity was also found in dense varicose fibres surrounding myenteric neurons (**Fig 3**, panels **E-F**). The percentage of BDNF-IR/CGRP-IR myenteric neurons in CTR preparations was 8.33±4.95% (n = 15) and significantly increased in preparations obtained from ABX-treated animals [40.52±4.08% (n = 16); p<0.0001]

### TrkB mRNA and protein levels in the mouse small intestine of control and ABX-treated mice

**Fig 4:**
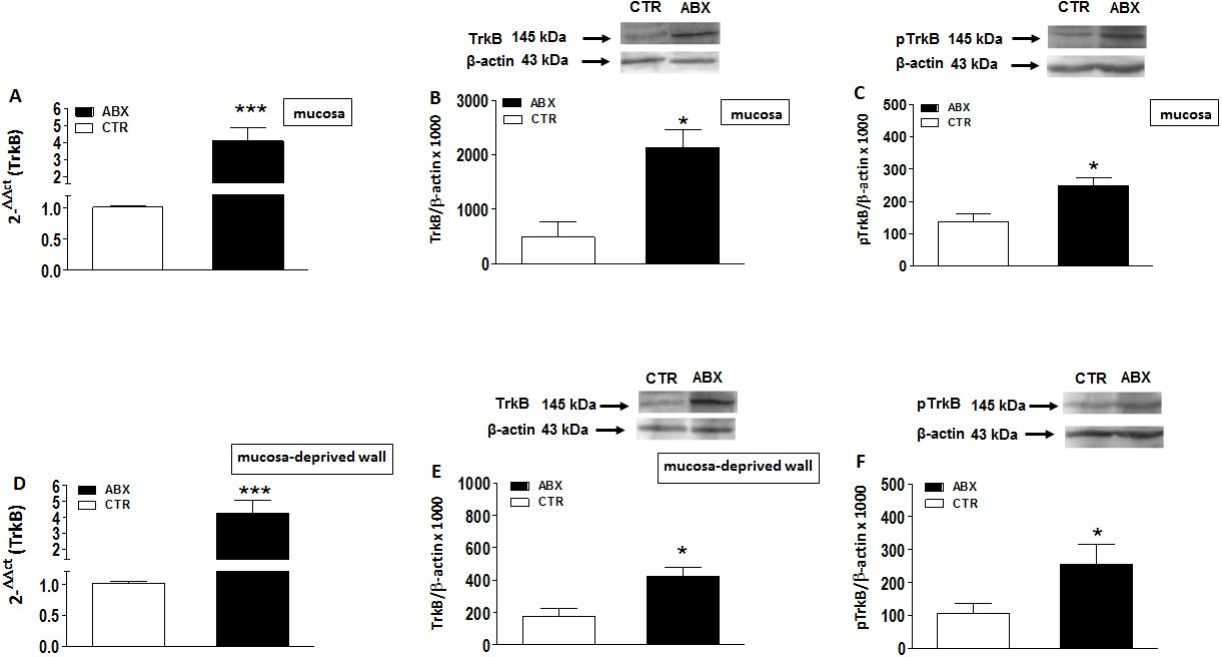
Expression of TrkB mRNA in juvenile mouse small intestine after antibiotic treatment-induced dysbiosis. (**A**, **D**) RT-PCR quantification of TrkB transcripts in preparations of the mouse small intestine mucosa (**A**) and mucosa-deprived whole wall (**D**) obtained from control (CTR, empty bars) and antibiotic treated mice (ABX, black bars). Values are mean±S.E.M. of 10 experiments of the percentage variation of relative gene expression with respect to values obtained in CTR animals. The relative gene expression was determined by comparing 2^-ΔΔCt^ values normalized to β-actin. Levels of expression of the native TrkB protein (**B**,**E**) and the phosphorylated active form at Tyr816 (pTrkB) (**C**,**F**) analyzed in the mucosa (**B**,**C**) and mucosa-deprived whole wall (**E**,**F**) of the mouse small intestine obtained from CTR (empty bars) and ABX-treated animals (black bars). Blots representative of immunoreactive bands for TrkB, pTrkB and β-actin in the different experimental conditions are reported on top of each panel. Samples (200 μg) were electrophoresed in SDS-8% polyacrylamide gels. Numbers at the margins of the blots indicate relative molecular weights of the respective protein in kDa. Values are expressed as mean±SEM of 5 experiments of the ratio x1000 of the optical density (O.D.) of TrkB vs β-actin in CTR and in ABX-treated preparations. *P<0.05 and ***P<0.001 vs CTR animals by unpaired Student’s t test.

TrkB mRNA levels in mucosa and mucosa-deprived whole intestinal wall specimens significantly increased (p<0.001 and p<0.0001, respectively) after antibiotic treatment as reported in **Fig 4,** panels **A** and **D**, respectively. In mouse small intestine preparations, western blot analysis of TrkB revealed a band at 145 kDa. In ABX-treated mice, TrkB protein levels significantly increased both in the mucosa (p<0.05) and in mucosa-deprived whole intestinal wall (p<0.05) (**Fig 4**, panels **B** and **E**). In both layers, the levels of TrkB phosphorylated on Tyr816 significantly increased (p<0.05) (**Fig 4**, panels **C** and **F**).

### Distribution of TrkB in small intestine LMMP whole-mount preparations from control and ABX-treated animals

**Fig 5:**
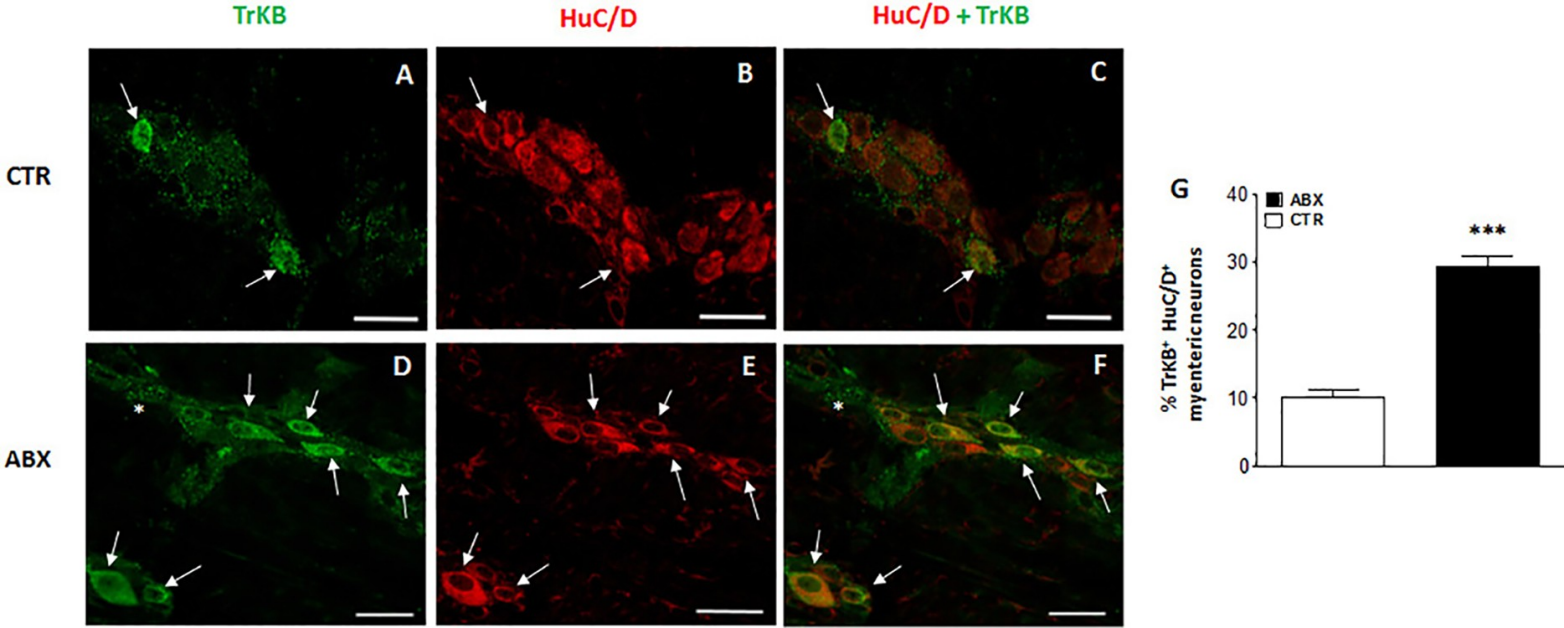
Effect of antibiotic treatment on TrkB distribution in juvenile mouse small intestine LMMP whole-mount preparations. Representative confocal microphotographs showing co-staining of TrkB (green) and HuC/D (pan-neuronal marker, red) in LMMPs obtained from CTR (**A-C**) and ABX-treated mice (**D-F**). In LMMPs obtained from both CTR and ABX-treated mice TrkB stained the soma of both large and medium neurons with an ovoidal shape (arrow). TrkB immunoreactivity was also found in discontinuous prolongations surrounding neurons and in the interconnecting fibres between ganglia (asterisk). In CTR preparations, TrkB faintly stained the soma of few neurons. After antibiotic treatment, the number of myenteric neurons staining for TrkB was significantly higher than in CTR (**D-E**). Bars: 50 μm. (**G**) Percentage of TrkB immunoreactive myenteric neurons in small intestine of CTR and ABX-treated mice. Values are given as mean ± SEM of at least 20 fields for each intestinal region. P<0.001 vs CTR animals by unpaired Student’s t test.

**Fig 6:**
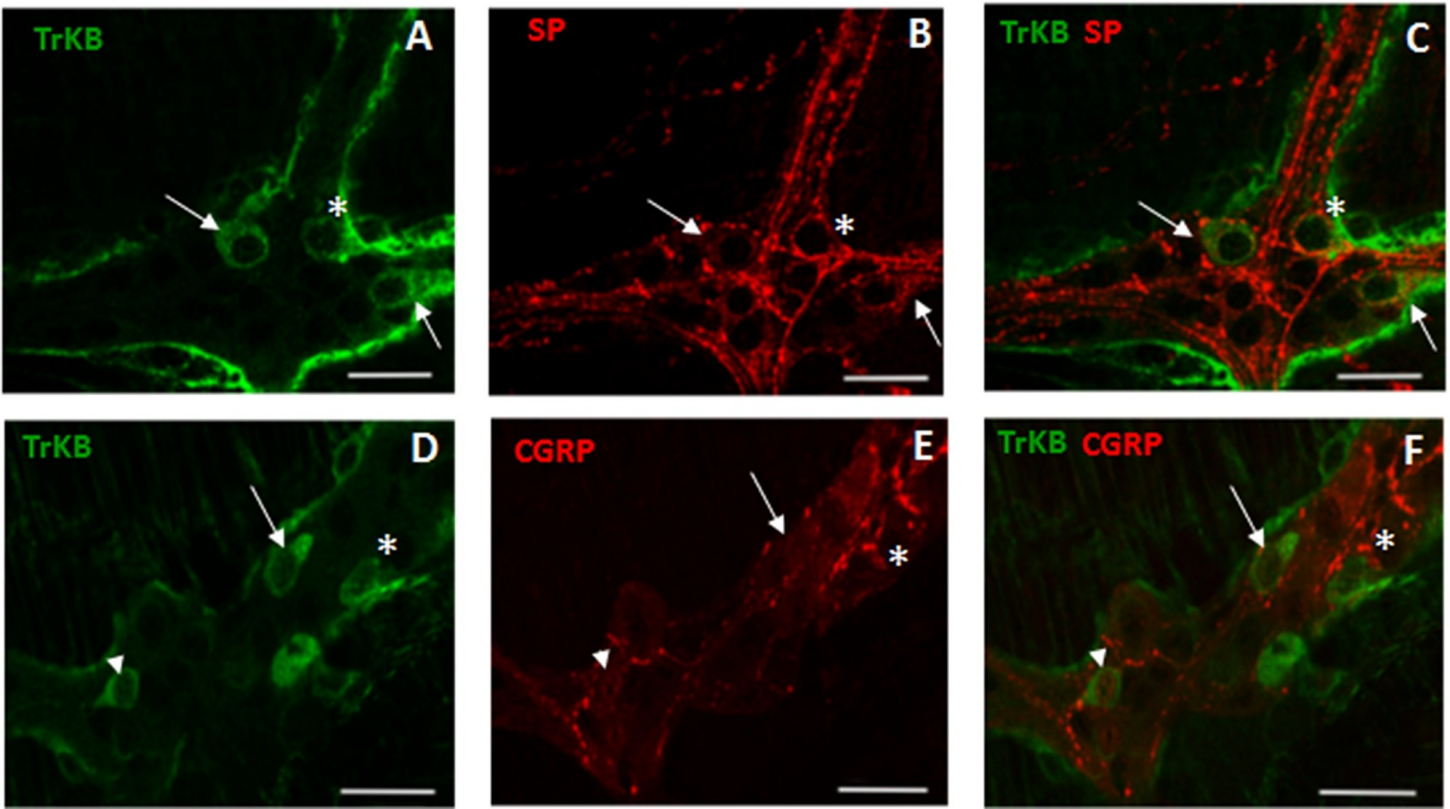
Relationship between TrkB, SP and CGRP immunoreactivity in juvenile mouse small intestine myenteric ganglia. (**A-C**) Representative micrographs of mouse small intestine LMMP whole-mount preparations obtained from ABX-treated mice showing co-localization of TrkB and SP. TrkB immunoreactivity was found in the soma of a population of SP-IR expressing neurons (arrows). Some TrkB myenteric neurons were surrounded by varicose SP-IR fibres (asterisk). (**D-F**) Representative micrographs of mouse small intestine LMMP whole-mount preparations obtained from ABX-treated mice showing co-localization of TrkB and CGRP. CGRP-IR was found in the soma (arrows) and nucleus (arrowhead) of TrkB-IR myenteric neurons. TrkB-IR myenteric neurons were surrounded by varicose CGRP-IR fibres (asterisk). Bars: 25 μμm.

In control small intestine LMMP whole-mount preparations, TrkB antibody stained few neurons (10.13±1.11%, n = 20) (**Fig 5**, panel **G**). As observed after co-staining with the pan neuronal marker, HuC/D, TrkB antibody stained the soma of large and medium-sized neurons with prevalently an ovoidal shape. In myenteric ganglia, TrkB immunoreactivity was also found in prolongations discontinuously surrounding neurons, in interconnecting strands between ganglia and in bundles of fibres running along the longitudinal smooth muscle (**Fig 5** panels **A-C** and **D-F**). After antibiotic treatment, a significant [29.40±1.58%, (n = 21) p<0.001) increase in the number of TrkB-IR myenteric neurons was observed with respect to preparations obtained from CTR mice (**Fig 5**, panels **D, F** and **G**). In whole-mount LMMP preparations obtained from both CTR and ABX-treated mice TrkB-IR neurons co-stained with SP (**Fig 6**, panels **A-C**). SP immunoreactivity was also detected in varicose fibres surrounding TrkB-IR myenteric neurons (**Fig 6**, panels **A-C**). The percentage of TrkB-IR neurons expressing SP in ABX-treated mice was not significantly different with respect to values obtained in CTR animals [14.81±4.45%, (n = 18); and 16.67±5.52% (n = 14), respectively; p = 0.79]. In both CTR and ABX-treated whole-mount preparations TrkB-IR myenteric neurons were also immunoreactive to CGRP (**Fig 6**, panels **D-F**). CGRP immunoreactivity was found in the soma and nucleus of TrkB-IR neurons as well as in varicose fibres surrounding TrkB-IR neurons (**Fig 6**, panels **D-F**). In CTR preparations, 16.67±5.69% (n = 16) of TrkB-IR myenteric neurons was immunoreactive to CGRP. This percentage significantly increased in preparations obtained from ABX-treated animals [42.86±4.68% (n = 20); p<0.001].

### BDNF and TrkB mRNA and protein levels in the hippocampus of antibiotic treated mice

**Fig 7:**
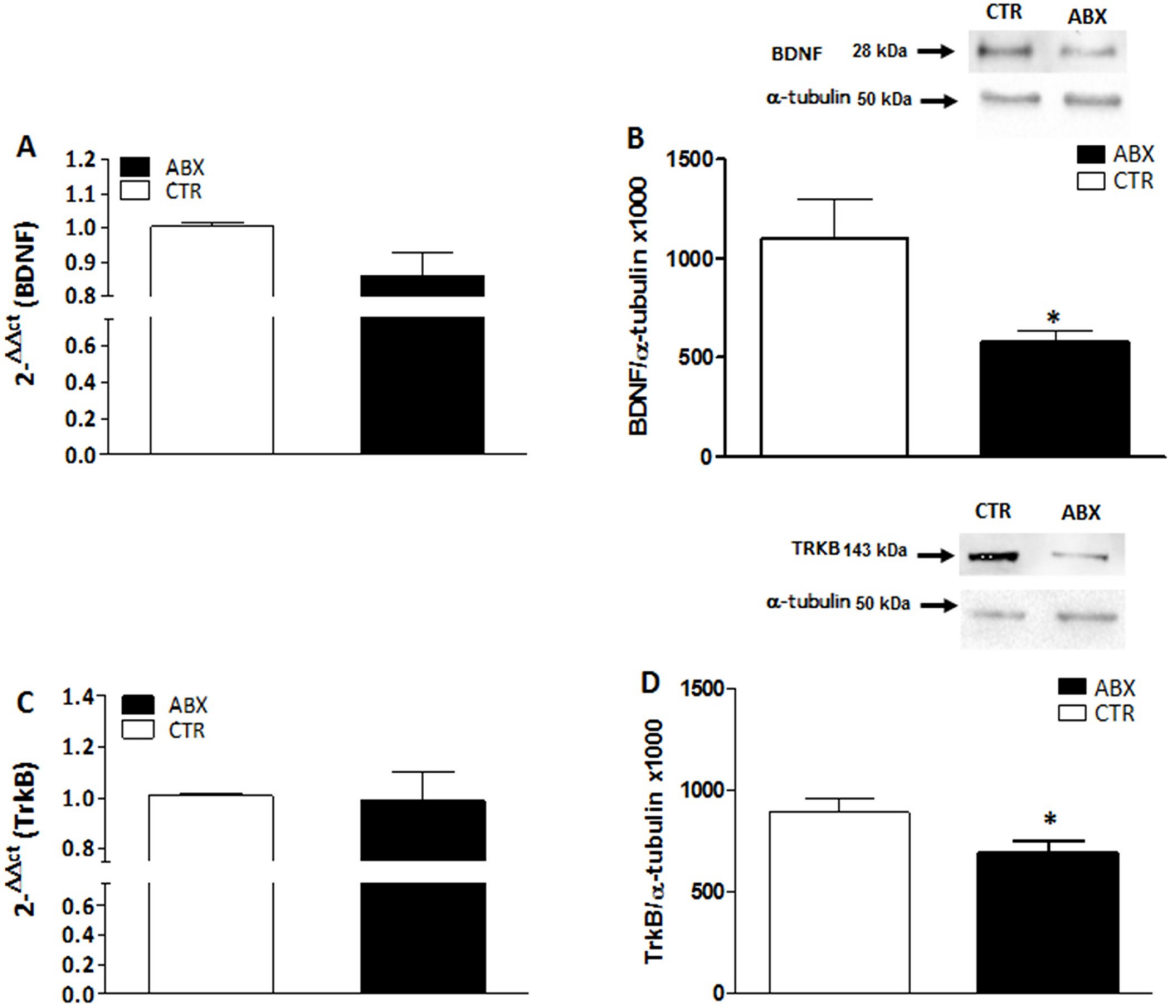
Expression of BDNF and TrkB in juvenile mouse hippocampus after antibiotic treatment-induced dysbiosis. (**A, C**) RT-PCR quantification of BDNF (**A**) and TrkB (**C**) transcripts in the mouse hippocampus obtained from control (CTR, empty bars) and antibiotic treated (ABX, black bars). Values are mean±S.E.M. of 5 experiments of the percentage variation of relative gene expression with respect to values obtained in CTR animals. The relative gene expression was determined by comparing 2^-ΔΔCt^ values normalized to β-actin. (**B, D**) BDNF (**B**) and TrkB (**D**) protein expression levels analyzed in the hippocampus obtained from CTR (empty bars) and ABX-treated animals (black bars). Blots representative of immunoreactive bands for TrkB and α-tubulin in the different experimental conditions are reported on top of each panel. Samples (200 μg) were electrophoresed in SDS-8% polyacrylamide gels. Numbers at the margins of the blots indicate relative molecular weights of the respective protein in kDa. Values are expressed as mean±S.E.M. of at least 5 experiments of the ratio x1000 of the optical density (O.D.) of BDNF and TrkB vs α-tubulin in CTR and in ABX-treated preparations. *P<0.05 vs CTR animals by unpaired Student’s t test.

**Fig 8:**
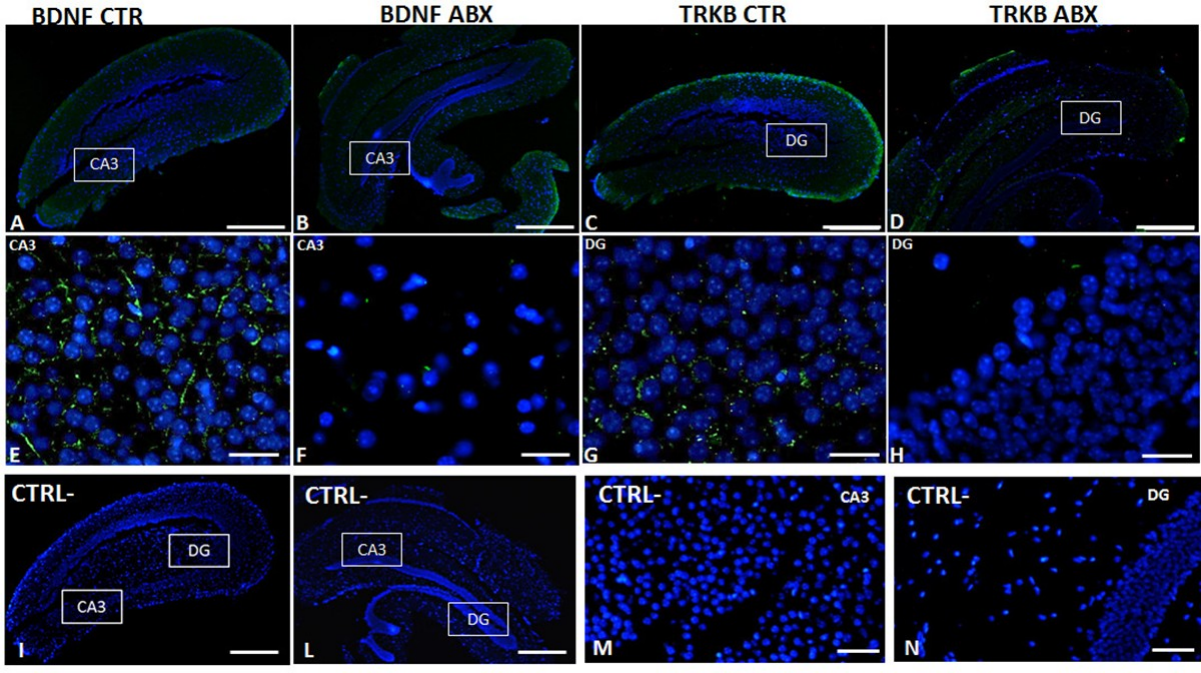
Distribution of BDNF and TrkB in juvenile hippocampus of CTR and ABX-treated mice. Fluorescence microscopy images showing green BDNF and TrkB immunostaining in panoramic images taken from CTR (**A,C**) and ABX-treated (**B,D**) mice, counterstained with DAPI (blue) (bars: 500 μm). Panels **E,F** represent higher magnifications of the CA3 subregion, showing a reduced immunostaining for BDNF (green) counterstained with DAPI (blue), in preparations obtained from ABX-treated mice (**F**) with respect to CTR (**E**) (bars: 25 μm). Higher magnification microscopy images showing green TrkB immunostaining in the dentate gyrus (DG) subregion of CTR (**G**) and ABX-treated (**H**) mice, counterstained with DAPI (blue) (bars: 25 μm). Panels **I, L, M, N** represent negative controls for each experimental group. (Bars in **I**, **L**: 500 μm; bars in **M**, **N**: 50 μm).

In the hippocampus of ABX-treated mice, BDNF and TrkB mRNA levels were unchanged (p = 0.071 and p = 0.876, respectively) with respect to CTR (**Fig 7** panels **A** and **C**). In contrast, both BDNF and TrkB protein levels were significantly lower in the hippocampus of ABX-treated mice (p<0.05 for both proteins) than in CTR (**Fig 7** panels **B** and **D**). To investigate the pattern of distribution of BDNF and TrkB, we performed fluorescent immunohistochemical staining for both proteins in hippocampal slices of CTR and ABX-treated mice. In the hippocampus of both CTR and ABX-treated animals, BDNF and TrkB stained the CA1-CA3 and dentate gyrus (DG) subregions. However, after ABX treatment, BDNF staining decreased in the CA3 subregion (**Fig 8**, panels **E**,**F**), while TrkB staining was reduced in the DG subregion (**Fig 8**, panels **G**,**H**).

### BDNF and TrkB mRNA and protein levels in the prefrontal cortex of antibiotic-treated mice

**Fig 9:**
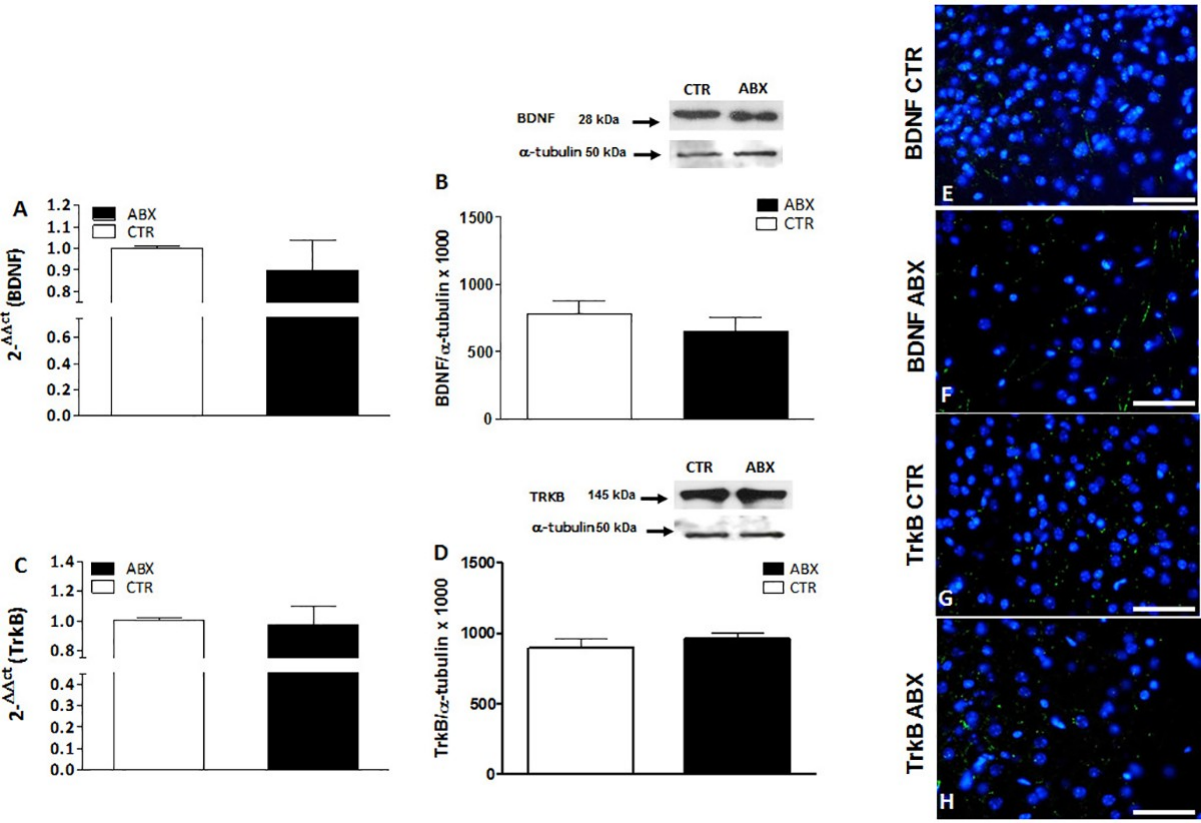
Expression of BDNF and TrKB in juvenile mouse prefrontal cortex (PFC) after antibiotic treatment-induced dysbiosis. (**A, C**) RT-PCR quantification of BDNF (**A**) and TrkB (**C**) transcripts in the mouse PFC obtained from control (CTR, empty bars) and antibiotic treated (ABX, black bars). Values are mean±S.E.M. of 7 experiments of the percentage variation of relative gene expression with respect to values obtained in CTR animals. The relative gene expression was determined by comparing 2^-ΔΔCt^ values normalized to β-actin. (**B, D**) BDNF (**B**) and TrkB (**D**) protein expression levels analyzed in the PFC obtained from CTR (empty bars) and ABX-treated animals (black bars). Blots representative of immunoreactive bands for BDNF and TrkB and α-tubulin in the different experimental conditions are reported on top of each panel. Samples (200 μg) were electrophoresed in SDS-8% polyacrylamide gels. Numbers at the margins of the blots indicate relative molecular weights of the respective protein in kDa. Values are expressed as mean±S.E.M. of 5 experiments of the ratio x1000 of the optical density (O.D.) of BDNF and TrKB vs α-tubulin in CTR and in ABX-treated preparations. (**E-H**) Fluorescence microscopy images showing immunostaining for BDNF (green, Panels **E**,**F**) and for TrkB (green, **G**,**H**) in the prefrontal cortex of CTR and ABX-treated mice counterstained with DAPI (blue). Bars: 50 μm.

In the prefrontal cortex of ABX-treated animals, BDNF and TrkB mRNA levels remained unchanged (p = 0.489 and p = 0.844, respectively) with respect to values obtained in CTR mice (**Fig 9**, panels **A** and **C**). BDNF and TrkB protein levels were also unchanged in the prefrontal cortex of ABX-treated mice (p = 0.37 and p = 0.45, respectively) (**Fig 9** panels **B** and **D**). Immunostaining for both BDNF and TrkB in the prefrontal cortex of ABX-treated mice did not display significant differences with respect to that observed in CTR animals (**Fig 9** panels **E-F**; **G-H**).

## Discussion

In this study, we provide evidence that a 2-week antibiotic treatment carried out in juvenile mice induces changes in the expression of BDNF and of its high affinity receptor, TrkB, in both the ENS and CNS. However, such alterations are different along the gut-brain axis, since BDNF and TrkB levels are up-regulated in the ENS, downregulated in the hippocampus and unmodified in the prefrontal cortex, suggesting that dysbiosis may predispose to regionally differentiated effects on BDNF signaling. The observed changes are closely superimposable to BDNF alterations observed in animal models of IBS and IBS patients alike, suggesting that an antibiotic treatment in adolescence may predispose to functional gut disorders later in life [13,39]. A two-week course of high‐dose, broad-spectrum, poorly-absorbable antibiotics is a cost‐effective model to characterize the many effects of gut dysbiosis on ENS and CNS structure and function, as shown previously by us and others [3,31,40,41]. However, we have recently reported that ABX treatment in juvenile mice determines a marked reduction in bacterial loads in mouse feces [41] and, consistent with previous observations, the faded commensal stimulation impacts gut neuromuscular function, brain anxiety and cognitive behaviors as well as key neuroimmune modulators of gut-brain dialogue in a manner comparable to that described in germ-free mice [42].

In analogy with the CNS, in the ENS, BDNF is a target-derived growth factor involved in neuronal development and survival during ontogenesis [15,43,44], but may also act as an excitatory neurotransmitter/neuromodulator, and is involved in the rearrangement of enteric neuronal circuitries, both in physiological conditions and in disease states, such as IBS [23,25,29]. This role is consistent with the presence of BDNF and TrkB immunoreactivity in juvenile mouse myenteric neurons, as previously demonstrated in the human and adult mouse gut [42,45]. In the small intestine of juvenile control mice, the number of myenteric neurons staining for both BDNF and TrkB was relatively low, and increased significantly after antibiotic treatment-induced dysbiosis. Accordingly, in mucosa-deprived intestinal wall segments of ABX-treated mice, both BDNF and TrkB mRNA and protein levels were up-regulated. Enhancement of BDNF and TrkB expression in the colonic *muscularis propria* containing the myenteric plexus was also observed in a rat model of chronic stress, inducing IBS symptoms [45]. We have recently demonstrated that in the juvenile mouse gut, microflora disruption is associated with important morphological and functional changes in the ENS [29]. From this standpoint, we cannot exclude that the increased expression of both BDNF and TrkB in the neuromuscular compartment may contribute to neuronal adaptation to dysbiosis. This hypothesis is strengthened by the demonstration of an augmented expression of the activated TrkB receptor, phosphorylated at Tyr816. BDNF, by binding to TrkB induces the receptor dimerization and autophosphorylation of tyrosine residues, including Tyr816, which is fundamental for BDNF-mediated structural and functional neuronal plasticity [46,47]. In the enteric microenvironment of the human and rodent gut, epithelial mucosal cells represent an important non-neuronal source of BDNF, playing a paracrine role by stimulating enteric reflexes, including peristalsis [24,25,45,48]. In the juvenile mouse small intestine mucosa, both BDNF and TrkB mRNAs and proteins were detected and their expression was upregulated after antibiotic treatment. High levels of BDNF and TrkB were found in the mucosal layer of mouse and rat models of IBS [25,45] as well as in the colonic mucosa of IBS patients [39]. In mice, intracolonic administration of fecal surnatants from IBS patients enhanced mucosal neurotrophin release and positively correlated with increased expression of TrkB and glial markers on mucosal enteric glial cells, which represent another source of BDNF in the gut [25]. Animals developed visceral hypersensitivity to colorectal distension, involving activation of an enteric glial cell-mediated connection between mucosal signals and enteric neurons via the BDNF-TrkB signaling. In our model, upregulation of enteric glial cells markers and alterations in the myenteric glial network was observed [31] suggesting the occurrence of a cross-talk between enteric glial cells and myenteric neurons, via BDNF-TrkB signaling, also in the small intestine of juvenile dysbiotic mice.

From a functional view point, BDNF exerts a facilitatory modulation on both motor and sensory gut functions via TrkB activation [27,49]. Administration of either human recombinant BDNF or a BDNF analog to both healthy human subjects and patients with chronic constipation accelerated colonic motility and increased stool frequency [23]. In the rat colon, endogenous BDNF favored peristalsis in a stimulus-dependent manner during mucosal stroking, and this effect was significantly reduced in heterozygotic (BDNF^+/-^) mice [24]. The excitatory effect of BDNF on gut motor function is mainly indirect, caused by the neurotrophin-induced release of other excitatory neurotransmitters, such as SP and CGRP [24,45]. This indirect action was substantiated by the evidence that addition of a monoclonal antibody to TrkB could reduce SP- and CGRP-induced contractions in the mouse ileum and distal colon longitudinal muscle [50]. Accordingly, in primary cultures of guinea pig myenteric plexus, BDNF promoted synaptic vesicle cluster density, via TrkB receptor activation, and enhanced the release of SP and CGRP [51]. In our study, as demonstrated in the rat colon [45], some populations of BDNF- or TrkB-immunoreactive myenteric neurons were also stained for SP, and SP-containing fibers were found in close proximity of neurons expressing BDNF and TrkB immunoreactivity. A similar co-staining was also found between subpopulations of BDNF-, TrkB- and CGRP-immunoreactive myenteric neurons. Overall, these observations are highly suggestive for the existence of a close interplay between the BDNF-TrkB signaling pathway and tachykinergic and CGRP-mediated neurotransmission, also in the juvenile mouse small intestine. The increased co-expression of BDNF and TrkB with both SP and CGRP observed after dysbiosis, further strengthens this hypothesis, although we cannot exclude that other neurotransmitter pathways may be related to perturbations of BDNF levels [17], such as glutamatergic NMDA receptors, which are highly expressed within the ENS [52,53]. Since the tachykinergic excitatory transmission to longitudinal smooth muscle of the juvenile mice small intestine is enhanced after chronic antibiotic treatment [29], we cannot exclude that such facilitation may be sustained, at least in part, by BDNF and TrkB signaling up-regulation. In agreement with this hypothesis, in a rat model of IBS, expression of endogenous BDNF and TrkB increased, and such enhancement favoured SP-mediated contractions of colonic circular muscle, contributing to the development of hypermotility [45].

As a neurotransmitter/neuromodulator, BDNF released from dorsal root ganglia plays a fundamental role in the modulation of chronic pain conditions caused by peripheral inflammatory responses [54]. Recently, BDNF was shown to modulate visceral hypersensitivity, which represents a crucial mechanism underlying visceral hypersensitivity in IBS. Visceral hypersensitivity is generated from disturbances in pain perception both in higher brain regions and in peripheral sensory pathways, including intrinsic primary afferents in the gut [52,55]. Heterozygous BDNF^+/-^ mice developed lower colonic nociception under both control conditions and after trinitrobenzesulfonic acid-induced colitis [29]. Enhanced BDNF expression in the mucosa of IBS patients significantly correlated with the degree of abdominal pain and with alterations in nerve fibers innervating the mucosa [39]. Co-localization of BDNF with CGRP, which is a marker of primary afferent neurons in the mouse small intestine [56], indicates that BDNF may also influence intrinsic primary afferent pathways modulating visceral sensitivity in the juvenile mouse small intestine. Interestingly, in primary cultures of guinea pig myenteric ganglia, BDNF is co-stored with CGRP neurons expressing the vanilloid receptor TRPV1, and is consistent with the co-localization of BDNF and CGRP in sensory neurons of the dorsal root ganglia [28,57].

Overall, the changes observed in enteric BDNF-TrkB signaling after antibiotic-induced microflora depletion in the juvenile mouse small intestine share many similarities with the alterations of BDNF signaling, previously observed in animal models of IBS and in IBS patients. IBS is a functional GI disease, displaying comorbidity with psychiatric disorders and is associated with anxiety and mood disorders [58]. In this study, we evaluated the occurrence of changes in BDNF and TrkB expression in two CNS regions, such as the hippocampus and prefrontal cortex, which are associated with development of altered behavioral patterns, stress responses and anxiety [9,59]. Several studies suggest that the GI microbiota may influence behavior by modulating BDNF production in the CNS. Young adult GF mice showed behavioral anomalies associated with altered levels of several biomarkers, including BDNF, in the amygdala and hippocampus and cingulate cortex [20,21]. Mice undergoing a massive antibiotic treatment from weaning onwards developed an altered behavioral phenotype characterized by impairment of cognitive functions later in life, which was paralleled by reduced BDNF levels in the hippocampus [22]. Accordingly, in this study, the same antibiotic treatment induced a reduction of BDNF and TrkB expression levels in the hippocampus suggesting an overall downregulation of the neurotrophin signaling pathway in this CNS region. In particular, a marked reduction of BDNF staining was observed in the CA3 subregion, while TrkB expression was lower in the DG subregion of dysbiotic mice. Interestingly, in a recent paper, CA3 pyramidal hippocampal neuron activity was significantly reduced in dysbiotic mice and correlated with reduced BDNF hippocampal levels [60]. These observations are all the more interesting, since, alterations in both the morphology and function of hippocampal CA3 pyramidal and DG granular neurons, as well as changes in the hippocampal BDNF-TrkB pathway, are associated with stress and depression [61,62]. In contrast, the antibiotic treatment did not modify BDNF and TrkB expression in the prefrontal cortex, as suggested by both biomolecular and immunohistochemistry data. These data may suggest that the hippocampus, more than the prefrontal cortex, represents a target region in microbiota-mediated modulation of brain development during adolescence [22]. Furthermore, our results suggest that changes in the saprophytic microflora have different consequences on the regulation of BDNF expression along the gut-brain axis. This region-specific tuning of BDNF expression may contribute to alter specific responses in the ENS (i.e. motility and sensitivity) and in the brain, fostering the development of GI disorders, characterized by psychiatric comorbidity ad alterations in the gut microbiota over time, such as IBS [63].

## Conclusions

In summary, the present data suggest that a dysbiosis induced by a massive antibiotic treatment during adolescence may alter BDNF and TrkB expression both in the ENS and CNS, although with different outcomes in the two nervous systems, later in life. We cannot exclude that these molecular changes may contribute to alter specific neurotransmitter pathways, involved in the development of functional gut diseases, such as IBS. These results further strengthen the concept that appropriate manipulation of the gut microbiome during adolescence, may reduce the probability of developing severe disorders that could affect the gut and the CNS later. This is all the more interesting in view of the worldwide increasing incidence of functional gastrointestinal disorders affecting the adolescent and young population [64,65].
